# Adenine methylation may contribute to endosymbiont selection in a clonal aphid population

**DOI:** 10.1186/1471-2164-15-999

**Published:** 2014-11-19

**Authors:** Victoria Reingold, Neta Luria, Alain Robichon, Aviv Dombrovsky

**Affiliations:** INRA/CNRS/UNSA University Nice Sophia Antipolis, 400 routes de Chappes, BP 167, Sophia Antipolis, 06903 France; Department of Plant Pathology, ARO, The Volcani Center, Bet Dagan, 50250 Israel

**Keywords:** *Acyrthosiphon pisum*, Bacteriocyte, Facultative/secondary bacterium, Epigenetic, Deoxyadenosine methylase (Dam)

## Abstract

**Background:**

The pea aphid *Acyrthosiphon pisum* has two modes of reproduction: parthenogenetic during the spring and summer and sexual in autumn. This ability to alternate between reproductive modes and the emergence of clonal populations under favorable conditions make this organism an interesting model for genetic and epigenetic studies. The pea aphid hosts different types of endosymbiotic bacteria within bacteriocytes which help the aphids survive and adapt to new environmental conditions and habitats. The obligate endosymbiont *Buchnera aphidicola* has a drastically reduced and stable genome, whereas facultative endosymbionts such as *Regiella insecticola* have large and dynamic genomes due to phages, mobile elements and high levels of genetic recombination. In previous work, selection toward cold adaptation resulted in the appearance of parthenogenetic *A. pisum* individuals characterized by heavier weights and remarkable green pigmentation.

**Results:**

Six adenine-methylated DNA fragments were isolated from genomic DNA (gDNA) extracted from the cold-induced *green* variant of *A. pisum* using deoxyadenosine methylase (Dam) by digesting the gDNA with the restriction enzymes *DpnI* and *DpnII*, which recognize the methylated and unmethylated GATC sites, respectively. The six resultant fragments did not match any sequence in the *A. pisum* or *Buchnera* genomes, implying that they came from facultative endosymbionts. The A1 fragment encoding a putative transposase and the A6 fragment encoding a putative helicase were selected for further comparison between the two *A. pisum* variants (*green* and *orange*) based on Dam analysis followed by PCR amplification. An association between adenine methylation and the two *A. pisum* variants was demonstrated by higher adenine methylation levels on both genes in the *green* variant as compared to the *orange* one.

**Conclusion:**

Temperature selection may affect the secondary endosymbiont and the sensitive Dam involved in the survival and adaptation of aphids to cold temperatures. There is a high degree of adenine methylation at the GATC sites of the endosymbiont genes at 8°C, an effect that disappears at 22°C. We suggest that endosymbionts can be modified or selected to increase host fitness under unfavorable climatic conditions, and that the phenotype of the newly adapted aphids can be inherited.

**Electronic supplementary material:**

The online version of this article (doi:10.1186/1471-2164-15-999) contains supplementary material, which is available to authorized users.

## Background

Due to their ability to alternate between sexual and parthenogenetic (asexual) modes of reproduction, their wing polyphenism and the complex relationship between their primary and secondary endosymbiotic bacteria, aphids have become an increasingly popular insect model for genomic, epigenetic, ecological, developmental and evolutionary studies. Complete genome sequences have become available for these insects, starting with the pea aphid *Acyrthosiphon pisum* [1, 2]. Moreover, the ability to generate a repertoire of variants with distinct behavioral and physiological traits within clonal (asexual) reproduction plays an important role in epigenetic studies [3, 4].

The pea aphid hosts different types of endosymbiotic bacteria within bacteriocytes that help it survive and adapt to new environmental conditions and habitats. Embryonic aphids acquire endosymbionts from their mothers before birth (viviparous) via a vertical-transfer mechanism [5, 6]. The primary endosymbiotic bacterium (*Buchnera aphidicola*) engages in obligate symbiosis with *A. pisum* [7–9], expressing crucial genes for the biosynthesis of essential amino acids (i.e., methionine, cysteine and tryptophan [9–11]); it also plays a role in aphid growth and reproduction [5, 12–14]. Due to its long host adaptation and suitability, the *B. aphidicola* genome is remarkably stable. There does not appear to have been any chromosome rearrangements or new gene acquisitions in the last 50 to 70 million years. The *B. aphidicola* genome is also characterized by a highly conserved gene order and is drastically reduced in size [10], contains only essential genes, that is, minimal regulatory proteins and almost no mobile elements. Therefore, it is no longer a source of new functional genes for adaptation of its host [15]. In contrast, the secondary endosymbionts are facultative bacteria that can provide the aphid host with resistance against fungal pathogens and parasites [16, 17], heat adaptation [18], host-plant specialization [19], manipulation of wing polyphenism under crowded conditions [20, 21], and delayed sexual development [22]. These secondary endosymbionts might induce morphological changes such as green pigmentation [23] and complete the nutritional role of the primary endosymbiont ([24], reviewed in [11]).

In contrast to the primary endosymbiont, the secondary endosymbionts usually contain large genomes, sometimes with phages and mobile elements, and exhibit higher rates of genetic recombination and mutation [13, 25]. For example, genomes of *Regiella* species, a known secondary endosymbiont, are similar in size to those of free-living bacteria, harbor active phages and plasmids, contain mobile elements and exhibit gene rearrangements [26].

The plasticity of the bacterial genome contributes to these microbes’ fascinating adaptation to various ecological niches and dramatic changes in the environment. A significant portion of the variability of the bacterial genome is due to transposable DNA elements [27], transposon movement, transposases, and accessory genes taken from hosts [28, 29], accompanied by the introduction of external chromosomal DNA (e.g., plasmids or phages) into the bacterium’s genome [29, 30]. These genomic changes play a crucial role in generating a broad spectrum of phenotypes [29] and may be associated with the adaptation of bacteria to their host-dependent lifestyle [13, 25].

A selection process for individual aphids adapted to a colder environment was carried out in our laboratory. This process generated two pea aphid variants: those exhibiting the typical orange pigmentation under optimal conditions (22°C) and those with unique green abdomen pigmentation at colder temperatures (8°C) [3, 31].

To date, there have been no reports on how endosymbionts might affect the epigenetic state (e.g., gene expression, DNA or histone modifications) of aphid genomes. The aim of this study was to investigate the role of endosymbionts in the phenotypic adaptation of aphids to unfavorable temperature conditions. We hypothesized that selection of a particular strain(s) of secondary endosymbiont and genome modifications such as adenine methylation might contribute to the adaptation of the selected aphid variant and cause phenotypic changes.

## Results

### Selection of an aphid variant with a unique pigmentation

**Figure 1:**
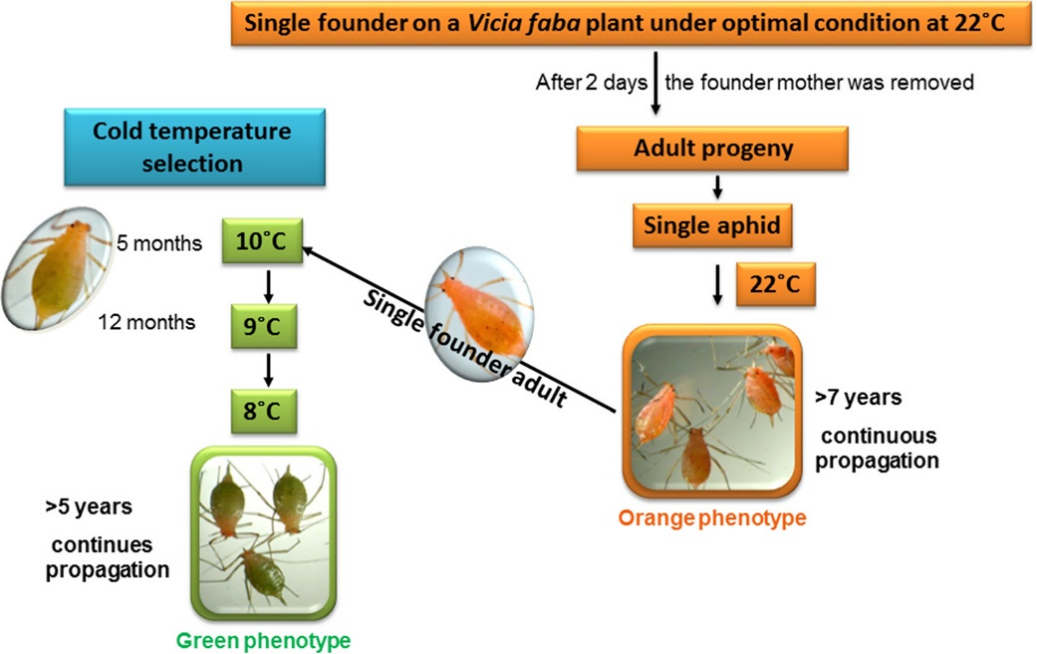
**Selection process used to generate the** ***green*** **variant.** Five to 10 orange adult *Acyrthosiphon pisum* (raised at 22°C) were placed on *Vicia faba* plants kept at 10°C. The aphid mothers were removed after 2 days, leaving the descendants. This process was repeated until a stable population emerged with a green phenotype (after 5 months). The original orange offspring did not reach maturation under these conditions. The population of cold-selected aphids with the green phenotype was propagated at 9°C for an additional year and then transferred to 8°C for continuous propagation (5 years).

In a previous study, selection for cold adaptation was performed by keeping ten orange adult aphids at 10°C for two days. After five months of this selection, we had obtained a viable and robust colony of *green* variants. To stabilize the cold-induced green variants, the selection pressure was increased in two steps: (1) the aphid population was propagated at 9°C for 1 year and (2) the temperature was then lowered to 8°C for continuous propagation (for 5 years; summarized in Figure 1).

The emerged *green* variants of *A. pisum* were characterized by a longer life cycle and slightly bigger bodies during the adult stage, with a heavier average weight [1.12 mg and 1.416 mg for the *green* adults (winged versus wingless, respectively); 0.99 mg and 1.36 mg for the *orange* adults (winged versus wingless, respectively)]. The *green* phenotype was heritable and robust when the aphids were kept at 8–10°C (Figure 1). Reversibility of the *green* phenotype back to *orange* was observed for all of the progeny without mortality when *green* mothers were placed back at 22°C. The emerged progeny were immediately *orange* with no process of phenotypic selection. These results showed unambiguously that, at 22°C, the pigments responsible for the *green* pigmentation are no longer synthesized and/or the corresponding enzymes are not induced. Genetic selection was observed during the passage from 22°C to 10°C and during the continued propagation at 9°C, and was maintained at 8°C. The enzymatic activity responsible for the change in color was inhibited when aphids were placed back at 22°C.

### Adenine methylation at the GATC sites of the secondary endosymbiont is temperature-dependent

**Figure 2:**
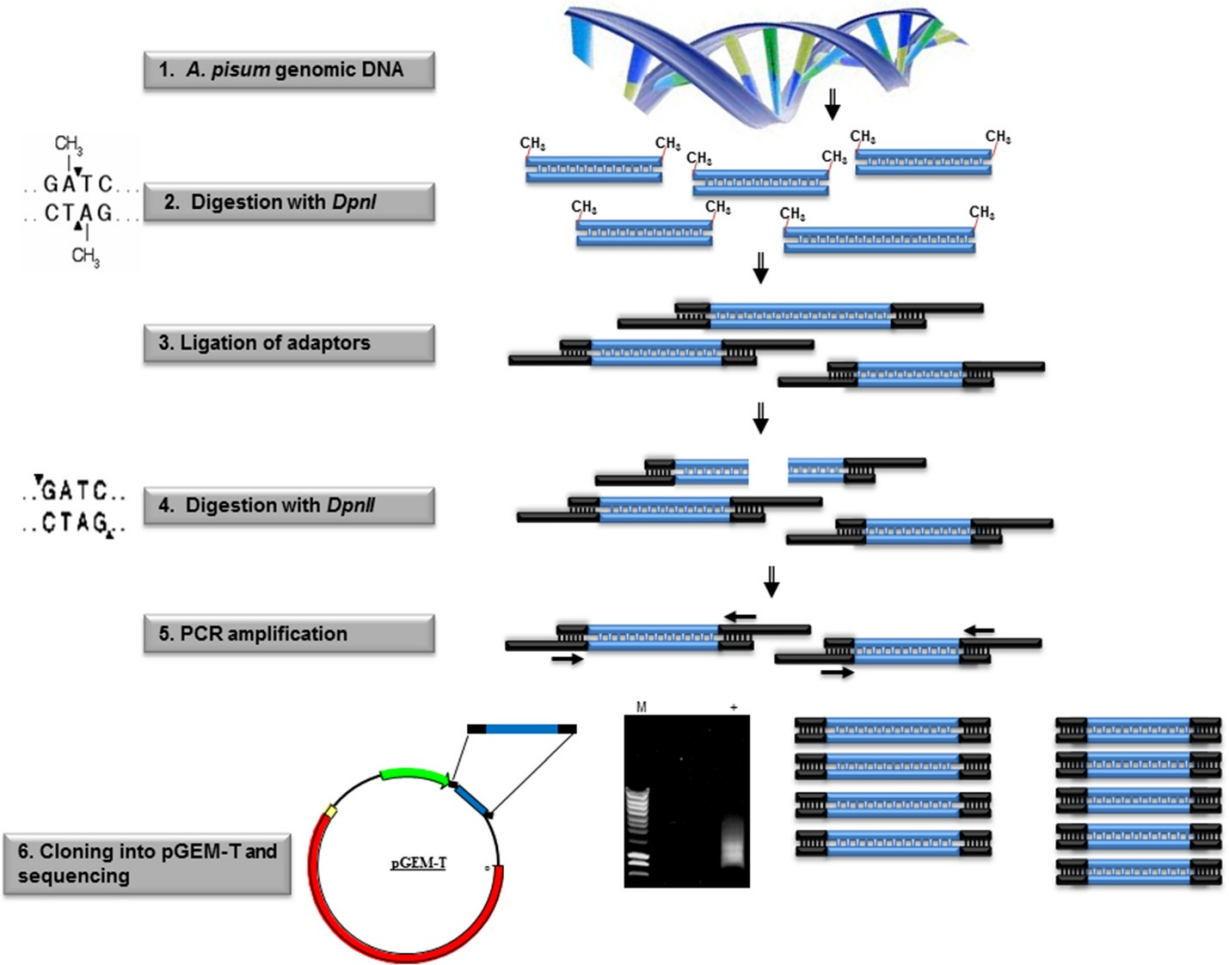
**Isolation of methyladenine genomic fragments using methyl-sensitive restriction enzymes, followed by PCR amplification.** DNA fragments containing methylated adenines were isolated from samples of total genomic DNA of *A. pisum* using the methylation-site-specific restriction enzyme *DpnI*, adaptor ligation and the methyl-sensitive restriction enzyme *DpnII*. This was followed by PCR amplification as described by Steensel and Henikoff [59]. The resulting PCR products were cloned and sequenced.

Six fragments were selected (A1–A6) from the adenine-methylated amplification procedure (Figure 2, Additional file 1: Table S1). BLAST analyses of the nucleotide and deduced amino acid sequences of the six fragments did not reveal any match to the genomes of the *A. pisum* aphid or the primary endosymbiont *B. aphidicola*. However, the results indicated that the sequences belong to the pea aphid facultative endosymbiont *R. insecticola* or other potential endosymbionts, such as those belonging to the *Yersinia* genus—well-known facultative endosymbionts of mealybugs (*Hemiptera*, *Coccoidea*, *Pseudococcidae*) [32] that were also detected in the BLAST analyses (Additional file 1: Table S1).

The A1 fragment encoded a hypothetical *R. insecticola* protein with a high level of sequence similarity to the transposase of *Microcystis aeruginosa* and the IS4 transposase of *Nostoc punctiforme*. Further BLAST-X analysis of the A1 fragment revealed a conserved transposase domain within the sequence (helix-turn-helix of the DDE superfamily endonuclease). The A2 and A4 fragments matched hypothetical proteins belonging to the genera *Regiella* and *Yersinia*. The A3 fragment was highly similar to the secG subunit of a translocase involved in protein export. Like the A1 fragment (transposase), the A6 fragment was also found to be highly similar to enzyme involved in DNA rearrangement in the endosymbionts (*Regiella* and *Yersinia*), the helicase gene. The A6 helicase was identified as highly conserved among bacterial species with 94%; 78% and 74% shared amino acid identity with *R. insecticola*, *Y. enterocolitica* and *Salmonella enterica*, respectively.

### Validation of adenine methylation at the GATC sites within the transposase gene

To confirm the variability of adenine methylation between *orange* and *green* variants, fragments A1 and A6 were selected for further analysis. Based on the nucleotide sequence of A1 in the *A. pisum* EST database (accession number EST-CV840801), three putative GATC sites of potential adenine methylation were identified within and upstream of the original A1 sequence (marked as A1M1–A1M3 in Additional file 2: Figures S1 and Additional file 3: Figure S2a). The A1 fragment (including the extended sequence obtained from the NCBI database based on WP_002757529.1 and YP_001863818.1) was used in a subsequent comparative study of the *green* and *orange* aphid variants.

**Figure 3:**
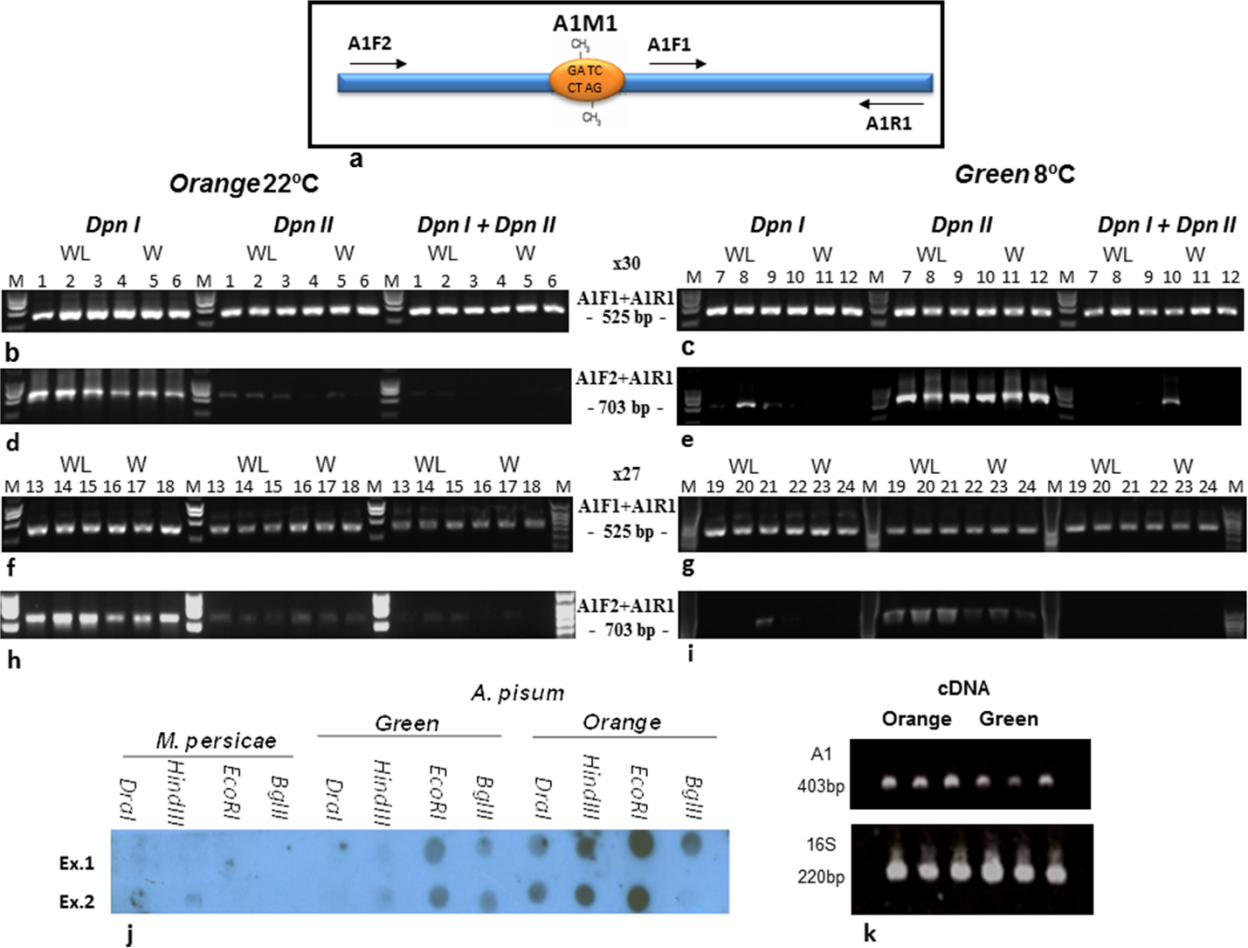
**Analysis of adenine methylation on the secondary endosymbiont’s transposase gene.** Schematic representation of the A1 fragment (*Regiella insecticola* transposase gene, accession number WP_006705102.1). Orange circle indicates the position of the examined adenine methylation site; arrows indicate primer positions and orientations **(a)**. PCR amplification of digested gDNA using A1F1 and A1R1 primers from *orange* and *green* variants (24 individual aphids) **(b-c** **and** **f-g)**. PCR using A1F2 and A1R1 primers on *orange* and *green* variants (24 individual aphids) **(d-e** **and** **h-i)**. Dot-spot hybridization (using A1-based DIG probe) on gDNA extracted from *A. pisum* (*green* and *orange* variants) and *Myzus persicae* **(j)**. Comparison of cDNA expression levels in the *orange* and *green* variants **(k)** of the A1 fragment (upper panel), and *R. insecticola* 16S (lower panel).

Genomic DNA (gDNA) was purified from *orange* and *green* adult aphids (42 individual aphids in total: 21 orange and 21 green—including 24 wingless and 18 winged aphids) and subjected to pre-digestion with *EcoRI* (keeping the A1 fragment intact). Each gDNA sample was divided into three subsamples and subjected to a further Dam digestion series with the two restriction enzymes *DpnI* and *DpnII*, separately and in combination. The digested gDNA served as a template for two PCR amplifications carried out using two primer pairs: A1F1, A1R1 (positive control, located downstream of the methylated site), and A1F2, A1R1, which flank the selected adenine-methylation site (A1M1; Figure 3a, Additional file 2: Figure S1).

For all 42 of the examined aphids (three samples of each—gDNA digested with *DpnI*, *DpnII* or a combination of the two), positive amplification was detected when the A1F1 and A1R1 primers were used in the PCR (positive control; Figure 3b-c). However, no amplicons were detected when the primers A1F2 and A1R1 were used together with the *DpnI*-digested gDNA from 13 of the *green* aphids, and medium to low amplicon levels were detected for the other 8. Amplification was detected in all of the tested *orange* aphids: a high level of amplicons for 17 of these aphids and a medium level of amplicons for the other 4. For the samples subjected to *DpnII* digestion, strong amplification was detected among the samples from *green* aphids, whereas low to zero amplification was observed among the samples from *orange* aphids (Figure 3b-i; Table 1). Moreover, no differences were detected between the winged and wingless *orange* and *green* aphids (Figure 3b-i; Table 1). The pattern of adenine methylation was also examined for two additional GATC sites (A1M2 and A1M3) clustered upstream of the transposase gene in most of the examined aphids (Additional file 2: Figures S1 and Additional file 3: Figure S2). The differences observed in amplification intensity within the A1M1–A1M3 sites in the two variants were not influenced by the transposase transcript levels (Figure 3k). Based on the Dam methylation amplification results, a clear pattern was detected that could be associated to the differences between the two aphid variants. However, to understand the causality and the mechanism involved, further study is required.

**Table 1:** **Summary of adenine methylation on the secondary endosymbiont’s transposase gene**

Primers		A1F & A1R1				A1F2 & A1R1	
Phenotype	*No. of aphids	*DpnI*	*DpnII*	*DpnI + DpnII*	*DpnI*	*DpnII*	*DpnI + DpnII*
**22°C (** ***Orange*** **)**	21	(21)+++	(21)+++	(21)+++	(17)+++ (4)++	(15) + (6)-	(5) + (16) -
**8°C (** ***Green*** **)**	21	(21)+++	(21)+++	(21)+++	(3)++ (5) + (13) -	(13)+++ (6)++ (2)+	(1)++ (3) + (17) -

*Number of individual *Acyrthosiphon pisum* aphids tested by PCR amplification. The amplicon intensity was categorized into three levels: **+++**, high; **++**, medium; +low; -, no amplification.

A digoxigenin (DIG) probe was used in dot-spot hybridization experiments to verify the presence of the A1 fragment (transposase) in gDNA obtained from *A. pisum* and *Myzus persicae*. The A1 DIG probe reacted specifically with the gDNA extracted from both *orange* and *green* variants of *A. pisum*, and failed to react with the gDNA of *M. persicae* (Figure 3j).

### Genome rearrangement by transposase gene

Two techniques—Southern blot and genome walking—were adopted to confirm the differential genomic pattern mediated by the secondary endosymbiont transposase. The transposase assumed to be involved in genome rearrangement events leading to the establishment of new *A. pisum* variants.

A differential amplification pattern was observed upon comparison of the two *A. pisum* variants using transposase-specific primers (GW-F or GW-R) combined with a random hexamer primer (N_5_) (Additional file 4: Figure S3a). Additional amplicons were detected in the *green* variant as compared to the *orange* variant (Additional file 4: Figure S3b), demonstrating the possibility of transposase movement to a new location in the endosymbiont genome. For further validation, two additional PCRs were performed: first, with forward and reverse random hexamer primers (without the addition of the transposase-specific primer, as a negative control), which gave a negative result (Additional file 4: Figure S3c, left panel); second, with increased annealing temperature allowing for better specificity, and resulting in enhancement of the variation (Additional file 4: Figure S3c, right panel). Additional distinction between the two *A. pisum* variants was obtained by Southern blot analysis using digested gDNA from the two variants and a specific probe for the transposase. The hybridization pattern in the *orange* variant was located on fragments with relatively higher molecular weight relative to the *green* variant (Additional file 4: Figure S3d).

### Validation of adenine methylation at GATC sites within the helicase gene

**Figure 4:**
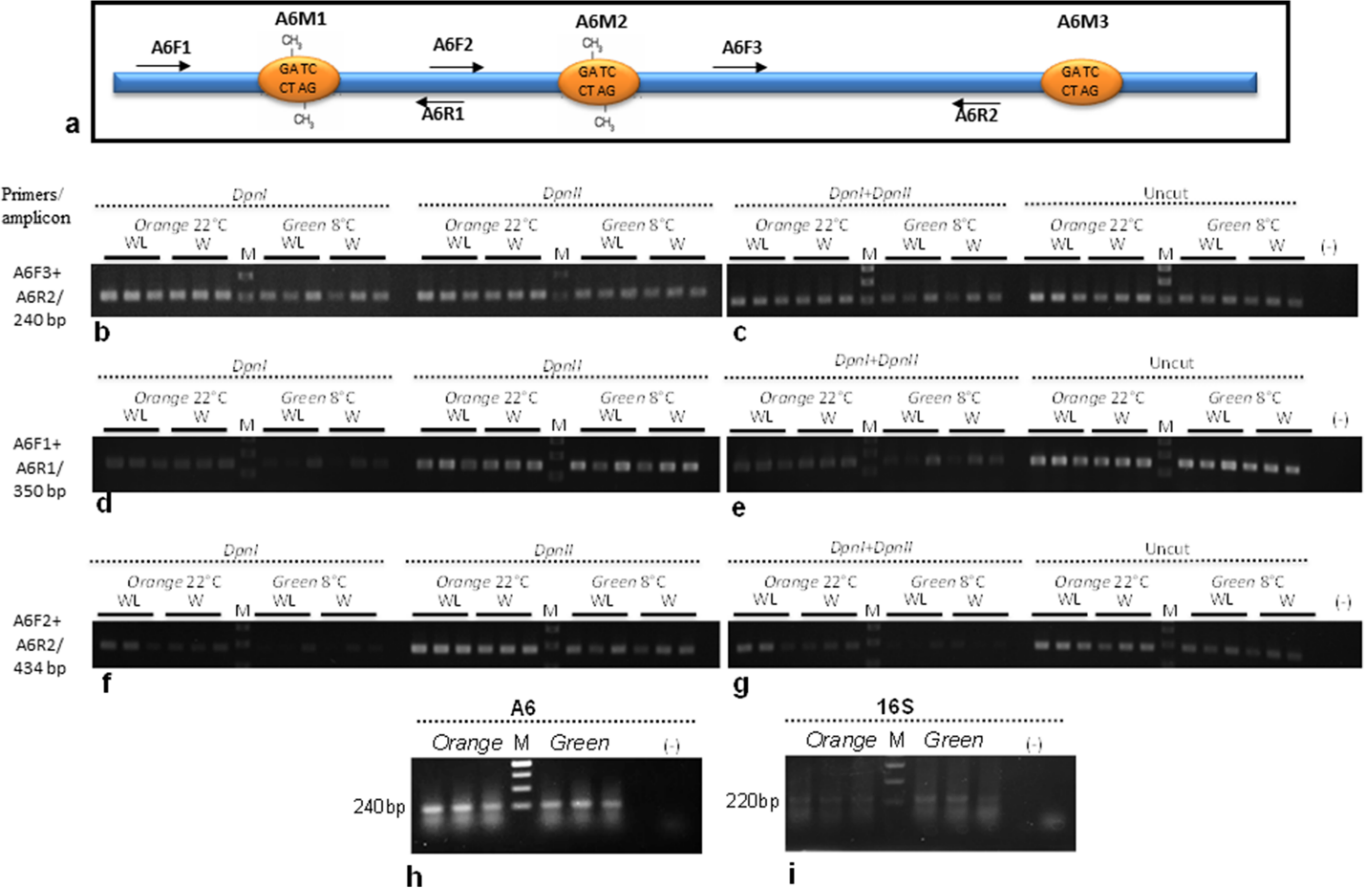
**Analysis of adenine methylation on the secondary endosymbiont’s helicase gene.** Schematic representation of the A6 fragment (*Regiella insecticola* helicase gene, accession number WP_006705384.1). Orange circles represent the positions of the examined adenine methylation sites (A6M1–A6M3); arrows indicate primer positions and orientations **(a)**. PCR amplification of digested gDNA from individual *A. pisum* aphids (*orange* and *green* variants) using A6F3 and A6R2 primers **(b-c)**, A6F1 and A6R1 primers **(d-e)**, and A6F2 and A6R2 primers **(f-g)**. Comparison of the cDNA expression levels in *orange* and *green* variants of the A6 fragment **(h)**, and *R. insecticola* 16S **(i)**.

A sequence analysis similar to that described above for the A1 fragment was applied to the extended nucleotide sequence of the A6 fragment obtained from the *R. insecticola* genome (94% identity; accession number WP_006705384.1). Three putative GATC sites were identified and further examined for adenine methylation (A6M1–A6M3; Figure 4a) using 12 *A. pisum* aphids (6 *orange* and 6 *green*). All tested aphids demonstrated a strong amplicon signal using A6F3 and A6R2 primers (positive control, no GATC site; Figure 4b-c and Table 2). Variation between the two aphid variants was detected within the A6M1 site by PCR amplification (using primers A6F1 and A6R1) of *DpnI*-digested DNA: strong amplicon intensity was obtained for the 6 *orange* aphids, whereas 5 *green* aphids demonstrated low to medium amplicon intensities, and no amplification was found for the 6th one (Figure 4d-e; Table 2). For the A6M2 site (A6F2 and A6R2 primers), medium to low amplification was detected for all 6 *orange* aphids, whereas medium amplification was detected for 3 *green* aphids and no amplification was detected for the other 3 (Figure 4f-g; Table 2). For the A6M3 site, high amplicon intensity with no variation was detected for the *orange* and *green* aphid variants (data not shown). The observed differences in adenine methylation on the A6M1 and A6M2 sites within the helicase gene were not related to gene expression (Figure 4h-i).

**Table 2:** **Summary of the adenine methylation on the secondary endosymbiont’s helicase gene**

Primers		A6F3 & A6R2			A6F1 & A6R1			A6F2 & A6R2		
Phenotype	*No. of aphids	*DpnI*	*DpnII*	*DpnI + DpnII*	*DpnI*	*DpnII*	*DpnI + DpnII*	*DpnI*	*DpnII*	*DpnI + DpnII*
**22°C (** ***Orange*** **)**	6	(6)+++	(6)+++	(6)+++	(6)+++	(6)+++	(6)+++	(2)++(4)+	(6)+++	(2)++(4)+
**8°C (** ***Green*** **)**	6	(6)+++	(6)+++	(6)++	(3)++(2)+	(6)+++	(3)++(3)+	(3)++	(6)+++	(2)+

*Number of individual *Acyrthosiphon pisum* aphids tested by PCR amplification. The amplicon intensity was categorized into three levels: +++, high; ++, medium; +low; -, no amplification.

## Discussion

Selection of aphids toward cold adaptation, as reported in a previous study [3], allowed us to obtain a viable and robust colony of *A. pisum* with green abdomen morphology (*green* variant) at 8–10°C [3]. The physiological changes induced by cold temperature were correlated with dramatic variations in cuticular proteins expression [3]. Variation in cuticle pigmentation has been linked to the lateral transfer of carotenoid production genes from fungi to their aphid hosts [33]. Extensive comparisons of the carotene metabolism of three *A. pisum* variants that differ in their cuticle pigmentation have been made [31].

The heritability of the *green* phenotype at 8–10°C [3], and the higher adenine methylation rates within the transposase and helicase genes (current study), suggest the existence of a coincidental double mechanism: strain selection and the regulation of new active genes by adenine methylation.

The combinatorial effects of a few thousand secondary endosymbionts in a limited number of bacteriocytes constitute an ideal mechanism for lateral gene transfer, allowing selection and amplification of singular rearranged genomes [12, 34]. This might support the assumption that the screening and selection process described by Dombrovsky *et al.* [3] recruited genes of endosymbiotic bacteria that are involved in host adaptation. These gene acquisitions, mediated by the temperature-dependent adenine methylation, might contribute to the ‘epigenetic’ regulation of unknown genes within the bacteria or the host genome.

In this study, we investigated the role of adenine methylation as an adaptive “on–off switch” [35] in endosymbiont bacteria, and whether this methylation is environmentally regulated in aphid endosymbionts and might thus be involved in the transition between *orange* and *green* phenotypes. Methylation at the N-6 position of adenine has been reported to be involved in many biological activities, from the control of chromosome replication and mismatch repair [36, 37] to the regulation of transcription, gene expression and virulence [38] and triggering pathogenicity by the reversible expression of surface antigens on bacterial membranes [39–41].

The rapid disappearance of the green pigmentation from cold-selected *green A. pisum* variants at 22°C suggests that temperature-dependent mechanisms control adenine methylation/demethylation within the endosymbiont genes, such as those encoding transposase and helicase. Adenine methylation of these genes may affect additional genes related to cold adaptation and the accumulation/disappearance of green pigment. Helicase is a key enzyme in DNA replication [42, 43] and mismatch repair [44, 45], which is mediated by adenine methylation in the bacterial genome [37, 44]. Interestingly, cold adaptation of *Bacillus subtilis* has been reported to be mediated by RNA-helicase [37], although the role of adenine methylation in the DNA-helicase gene remains unknown.

A similar temperature-dependent phenomenon involving transposase activity in plants has been reported to be mediated by adenine methylation [46]. Moreover, transposases of the THAP (P transposase) superfamily have been shown to be involved in cell-cycle regulation (in *Caenorhabditis elegans* [47] and different fish species [48]), epithelial cell regulation (in humans [49, 50]) and the regulation of gene expression and plant development [51]. Transposases from this superfamily are also essential for the development of *Oxytricha*, a genus of ciliate protozoa, in which they appear to rearrange hundreds of thousands of DNA pieces to form functional rearranged genes. The organism inactivates the transposases when they are no longer required, resulting in a stable genome architecture [52]. Furthermore, a drastic effect of adenine methylation on transposase activity has been reported in *Escherichia coli* [53].

As in other organisms, transposase genes in the endosymbiont bacteria of aphids might also be involved in genome rearrangement. This mechanism might help minimize the time required for the host to adapt to new environmental conditions [54, 55]. The transcription start time [35] and location of the transposase within the genome of the aphid endosymbiont bacteria might be controlled by adenine methylation within and upstream of the gene, as has been shown for other genes whose promoters exhibit Dam methylation [37, 53, 56]. Selective activation of the transposase within the bacteria may, in turn, affect the expression of essential genes and thereby contribute to the adaptation of the endosymbiont and its host to new environments, as described in this study for cold adaptation. More experiments should be performed in aphids to further evaluate the role of adenine methylation in transposases and other genes of secondary endosymbionts; this might be attained by high-throughput methods.

We can hypothesize that at least two methylation mechanisms coexist in aphids and provide epigenetic-based adaptive capabilities. The primary mechanism functions at the aphid genome level and involves the methylation of cytosine [3, 4, 57, 58]. The secondary mechanism—the cooperative complementary mechanism described in this study—is based on the regulation of adenine methylation within “dynamic” endosymbiont genomes and enables the development of host aphid phenotypes that are adapted to a given environment. This paper suggests a theory in which the involvement of bacterial adenine methylation, as an ‘epigenetic regulator’, helps aphids adapt to extreme environmental conditions and host phenotypic diversity.

Our experimental data demonstrate that the selection of rare profiles in a repertoire of strains is correlated with adenine methylation of pre-selected key proteins, which have the potential to rearrange the endosymbiont genome and lead to the development of a viable and robust aphid phenotype in an unfavorable environment.

## Conclusion

Our findings suggest that secondary endosymbionts contribute to aphid adaptation via the Dam mechanism. Regulation of adenine methylation within ‘dynamic’ endosymbiont genomes (e.g., transposase and helicase genes) is an additional mechanism assisting in aphid adaptation to a given environment which, in some cases, can be accompanied by a visible morphological phenotype. Transposase-mediated transposition may play a role in the multiple genetic and epigenetic mechanisms that together contribute to aphid phenotypic criteria and might act in concert to maximize host adaptation to unfavorable environmental conditions.

## Methods

### Maintenance and propagation of aphid species and variants

Pea aphids (*A. pisum*) were maintained on *Vicia faba* plants grown in cages in a propagation chamber kept at 22 ± 3°C and 60% relative humidity with a light/dark photoperiod of 16/8 h. The selection of aphid variants at lower temperatures was carried out in an environmental test chamber (Sanyo, Bensenville, IL, USA) in which the temperature, humidity and photoperiod were kept at 8°C, 60% and 16 h light/8 h dark, respectively. The green peach aphid *M. persicae* (Sulzer) was raised on mustard plants (*Brassica perviridis* cv. Tendergreen) in a greenhouse kept at 25 ± 3°C.

### Phenotypic selection at cold temperatures

Five to ten adult *orange* aphids (raised at 22°C) were placed in a container kept at 10°C and the founder aphids were removed after 2 days. This process was repeated for 5 months, until a stable and robust population with a green abdomen phenotype was established. The population of cold-selected aphids (*green* phenotype) was propagated at 9°C for an additional year and the ambient temperature was then lowered to 8°C for continuous propagation (>5 years) (Figure 1).

### Detection of methyl-adenine

Adenine-methylated DNA fragments were isolated from *A. pisum* gDNA (*green* variants). The fragments were identified and isolated using a pair of restriction enzymes recognizing the same nucleotide sequence (GATC): the first, *DpnI*, digests while the adenine is methylated; the second, *DpnII*, is blocked by the presence of a methyl group on the adenine. This procedure is described in detail in Figure 2. Briefly, gDNA was extracted from 100 mg of *A. pisum* (*green* and *orange* variants) using the DNeasy Plant Mini Kit (Qiagen, Hilden, Germany). DNA fragments containing methylated adenines were isolated from the genomic DNA using the methylation-specific restriction enzyme *DpnI* together with the methyl-sensitive restriction enzyme *DpnII* (New England Biolabs, Ipswich, MA, USA). The digested fragments were ligated to the adaptors:

AdRt (5′-CTAATACGACTCACTATAGGGCAGCGTGGTCGCGGCCGAGGA-3′) and AdRb (5′-TCCTCGGCCG-3′). This was followed by PCR amplification using AdR_PCR (5′-GGTCGCGGCCGAGGATC-3′), as described previously [59]. The resulting PCR products were cloned into pGEM-T Easy (Promega, Madison, WI, USA) and sequenced from both directions (T7 and SP6) (Figure 2).

### Detection of adenine methylation within the transposase gene obtained from an individual aphid

Total DNA was extracted from individual *A. pisum* aphids (Qiagen) propagated at 20°C (*orange* variant) or 8°C (*green* variant). The obtained gDNA was pre-digested with *EcoRI* and then re-digested with *DpnI, DpnII* or a combination of the two enzymes (each reaction performed separately). The digested gDNA was then PCR-amplified using two pairs of primers: A1R1 (5′-TGTCATGACGTCGACCATTT-3′) paired with the primers A1F1 (5′-TAGTGGGGCTATCGTTGGAA-3′), A1F2 (5′-TTTAAGATTCCGCCTGGTTG-3′) and A1F3 (5′-GATTATCATGGCAGCGCATA-3′), which were designed for the transposase gene (Figure 3a).

Expression differences between the *orange* and *green* variants were examined by extracting total RNA from single aphids. This RNA (50 μl) served as the template for cDNA synthesis using oligo-dt and random hexamer as the reverse primer. The cDNA was later amplified for 26 cycles using a specific primer set for the coding region of the transposase gene (as forward primer: 5′-TTTTTACCAACCCCATTGGA-3′ and reverse primer R1) and the 16S gene (as forward primer: 5′-ATCGGGGAGTAGCTTGCTAC-3′ and reverse primer: 5′-CTAGAGATCGTCGCCTAGGTA-3′) belonging to *Regiella*.

### Detection of adenine methylation within the helicase gene obtained from an individual aphid

Total DNA from 24 *A. pisum* aphids (12 from each variant—*orange* and *green*) was extracted and treated as described above. The digested DNA was PCR-amplified using three primer sets: for a positive control amplification (including no restriction sites), A6F3 (5′-TTGCATGAACCCATGACATT-3′) and A6R2 (5′-AATGCCGACATAAGCCAAAC-3′) were used to amplify the 240-bp amplicon. For the detection of methylation on site A6M1, A6F1 (5′-GTATCGAGGAAATCACCAAGC-3′) and A6R1 (5′-GCATAATTTCTGCCATCCAG-3′) were used to amplify the 350-bp amplicon; and for the detection of methylation on site A6M2, A6F2 (5′-TTACTCACTGGATGGCAGAAA-3′) and A6R2 were used to amplify the 434-bp amplicon (Figure 4a).

### Genome walking for transposase gene

To examine the possible occurrence of genome rearrangement, a ‘genome walking’ strategy was selected. Briefly, *EcoRI*- and *BamHI*-digested gDNA extracted from *green* and *orange* variants served as the templates for PCR amplification using a single specific primer located at the extremities of the extended A1 fragment (transposase gene) (GW-F: 5′-AGTGGGTTTTCTCTCACTGAGT-3′ or GW-R: 5′-CAACCAGGCGGAATCTTAAAC-3′) combined with a random hexamer primer (Additional file 4: Figure S3a). The reaction conditions were as follows: 95°C for 2 min followed by 35 cycles of 95°C for 30 s, 42°C for 30 s, 72°C for 1.5 min and a final elongation at 72°C for 5 min. The resultant PCR products were separated on a 1% agarose gel and selected fragments were cloned into pGEM-T Easy followed by nucleotide sequencing. Additional PCRs were carried out by increasing the annealing temperature (5 cycles at 42°C and 30 cycles at 50°C), and a negative control on the digested gDNA using random hexamer solely.

### DIG labeling and hybridization

Part of the A1 fragment (400 bp, transposase gene) was used for probe preparation. DIG-labeled nucleotides were introduced into the probe by PCR amplification. The reaction mixture contained the forward primer A1F1, the complementary primer A1R1 and a dNTP mixture containing DIG-labeled dUTP (2 mM dATP, dCTP, dGTP, 1.3 mM dTTP and 0.7 mM alkali labile DIG-11 dUTP), 100 pM DNA template and 1.5 units of Dream Taq polymerase (Fermentas-Thermo Fisher Scientific, Burlington, Canada).

### Dot blot

Each gDNA sample (two gDNA samples from each of the following: *M. persicae* and the *orange* and *green* variants of *A. pisum*) was digested separately with four restriction enzymes (*BglII*, *EcoRI*, *DraI* and *HindIII*). A 2-μl aliquot (100 ng) of each sample was blotted on a positively charged membrane (Roche, Basel, Switzerland) and then cross-linked under UV for 3 min.

### Southern blot

Samples (5 μg) of gDNA extracted from *orange* and *green* variants were digested with *EcoRI* and *BamHI* and separated on a 0.7% agarose gel. Then transferred to a positively charged membrane and cross-linked under UV.

Both membranes were pre-hybridized at 50°C in DIG Easy Hyb solution (Roche). Hybridization was performed at 50°C overnight followed by a high-stringency wash. A CSPD chemifluorescence kit (Roche) was used to detect the probe signal on X-ray film.

## Electronic supplementary material

Additional file 1: Table S1: Sequences and BLAST results for the adenine methylated fragments. (DOCX 30 KB)

Additional file 2: Figure S1: The nucleotides sequence and amino acid prediction of the aphid *Transposase* gene (A1 fragment) The DNA fragment (A1) was isolated from *A. pisum* based on methylation of adenine. **(a)** The nucleotide sequence of the original DNA fragment and the deduced amino acid encodes for a transposase gene. The translated sequence matches the transposase of: *Microcystis aeruginosa* (accession number WP_002757529.1) and *Nostoc punctiforme* (accession number YP_001863818.1). In gray the two putative sites for methylation on adenine flanking the region. **(b)** The EST-CV840801, represents a DNA fragment from the *A. pisum* EST data bank that shows high sequence similarity to the A1 DNA fragments. The upper lines are the nucleotide sequence encoding for transposase, and below is the deduced amino-acid residues. In bold, the transposase coding region. Mark in gray, the three putative sites for methylation on adenine that were identified upstream to the transposase gene. The primers sequences underlined and labeled in bold. (DOC 42 KB)

Additional file 3: Figure S2: Extended analysis of adenine methylation in the transposase gene. Total DNA was extracted from each individual *A. pisum* aphid propagated at 20°C (*orange* variant, lanes 1–3) or at 8°C (*green* variant, lanes 4–6). The obtained gDNA was pre-digested with *EcoRI*, and then re-digested by *DpnI*, *DpnII* or both. **(a)** Schematic representation of the extended A1 fragment (transposase gene). Orange circles represent the potential adenine methylation sites and arrows indicate the primers positions. The digested gDNA was amplified by PCR using three pairs of primer: A1R1 combined with **(b)** A1F1, **(c)** A1F2 or **(d)** A1F3. (TIFF 122 KB)

Additional file 4: Figure S3: Genome rearrangement. **(a)** Schematic representation of the extended A1 fragment (transposase gene) and the method used. Restriction enzymes indicated at the top, arrows indicates primer position and orientation. Orange circles represent the potential adenine methylation sites (A1M1-A1M3). **(b)** Differential amplification pattern on digested gDNA obtained from *orange* (O) and *green* (G) aphids. PCRs carried out using singular transposase specific primer [GW-F (left panel) or GW-R (right panel)] combined with random hexamer (N_5_), (-) indicates negative control, without template. **(c)** Controls: random hexamer solely (left panel) and increasing the annealing temperature in order to increase the specificity of the transposase specific primer (right panel). **(d)** Southern blot analysis on *orange* or *green* aphid variants digested gDNA (*EcoRI* or *BamHI*), the transposase DIG-probe (400 bp) reacted positively with the unlabeled amplicon (positive control, right lane). (TIFF 357 KB)

## References

[1] Brisson JA, Stern DL (2006). The pea aphid, *Acyrthosiphon pisum*: an emerging genomic model system for ecological, developmental and evolutionary studies. Bioessays.

[2] Consortium IAG (2010). Genome sequence of the pea aphid *Acyrthosiphon pisum*. PLoS Biol.

[3] Dombrovsky A, Arthaud L, Ledger TN, Tares S, Robichon A (2009). Profiling the repertoire of phenotypes influenced by environmental cues that occur during asexual reproduction. Genome Res.

[4] Walsh TK, Brisson JA, Robertson HM, Gordon K, Jaubert-Possamai S, Tagu D, Edwards OR (2010). A functional DNA methylation system in the pea aphid, *Acyrthosiphon pisum*. Insect Mol Biol.

[5] Moran N, Wernegreen J (2000). Lifestyle evolution in symbiotic bacteria: insights from genomics. Trends Ecol Evol.

[6] Sandstrom JP, Russell JA, White JP, Moran NA (2001). Independent origins and horizontal transfer of bacterial symbionts of aphids. Mol Ecol.

[7] Moran NA, Telang A (1998). Bacteriocyte-associated symbionts of insects - a variety of insect groups harbor ancient prokaryotic endosymbionts. Bioscience.

[8] Moran NA, Baumann P (2000). Bacterial endosymbionts in animals. Curr Opin Microbiol.

[9] Shigenobu S, Wilson AC (2011). Genomic revelations of a mutualism: the pea aphid and its obligate bacterial symbiont. Cell Mol Life Sci.

[10] Moran NA, Plague GR, Sandstrom JP, Wilcox JL (2003). A genomic perspective on nutrient provisioning by bacterial symbionts of insects. Proc Natl Acad Sci U S A.

[11] Brinza L, Vinuelas J, Cottret L, Calevro F, Rahbe Y, Febvay G, Duport G, Colella S, Rabatel A, Gautier C, Fayard JM, Sagot MF, Charles H (2009). Systemic analysis of the symbiotic function of *Buchnera aphidicola*, the primary endosymbiont of the pea aphid *Acyrthosiphon pisum*. C R Biol.

[12] Braendle C, Miura T, Bickel R, Shingleton AW, Kambhampati S, Stern DL (2003). Developmental origin and evolution of bacteriocytes in the aphid-Buchnera symbiosis. PLoS Biol.

[13] Dale C, Moran NA (2006). Molecular interactions between bacterial symbionts and their hosts. Cell.

[14] Baumann P, Baumann L, Lai CY, Rouhbakhsh D, Moran NA, Clark MA (1995). Genetics, physiology, and evolutionary relationships of the genus *Buchnera*: intracellular symbionts of aphids. Annu Rev Microbiol.

[15] Tamas I, Klasson L, Canback B, Naslund AK, Eriksson AS, Wernegreen JJ, Sandstrom JP, Moran NA, Andersson SG (2002). 50 million years of genomic stasis in endosymbiotic bacteria. Science.

[16] Scarborough CL, Ferrari J, Godfray HCJ (2005). Aphid protected from pathogen by endosymbiont. Science.

[17] Oliver KM, Russell JA, Moran NA, Hunter MS (2003). Facultative bacterial symbionts in aphids confer resistance to parasitic wasps. Proc Natl Acad Sci U S A.

[18] Montllor CB, Maxmen A, Purcell AH (2002). Facultative bacterial endosymbionts benefit pea aphids *Acyrthosiphon pisum* under heat stress. Ecol Entomol.

[19] Tsuchida T, Koga R, Fukatsu T (2004). Host plant specialization governed by facultative symbiont. Science.

[20] Muller CB, Williams IS, Hardie J (2001). The role of nutrition, crowding and interspecific interactions in the development of winged aphids. Ecol Entomol.

[21] Leonardo TE, Mondor EB (2006). Symbiont modifies host life-history traits that affect gene flow. Proc Biol Sci.

[22] Miura T, Braendle C, Shingleton A, Sisk G, Kambhampati S, Stern DL (2003). A comparison of parthenogenetic and sexual embryogenesis of the pea aphid Acyrthosiphon pisum (Hemiptera: Aphidoidea). J Exp Zool B Mol Dev Evol.

[23] Tsuchida T, Koga R, Horikawa M, Tsunoda T, Maoka T, Matsumoto S, Simon J-C, Fukatsu T (2010). Symbiotic bacterium modifies aphid body color. Science.

[24] Perez-Brocal V, Gil R, Ramos S, Lamelas A, Postigo M, Michelena JM, Silva FJ, Moya A, Latorre A (2006). A small microbial genome: the end of a long symbiotic relationship?. Science.

[25] Anderson JO, Anderson SGE (1999). Insights into the evolutionary process of genome degradation. Curr Opin Genet Dev.

[26] van der Wilk F, Dullemans AM, Verbeek M, van den Heuvel JF (1999). Isolation and characterization of APSE-1, a bacteriophage infecting the secondary endosymbiont of *Acyrthosiphon pisum*. Virology.

[27] Kleckner N (1981). Transposable elements in prokaryotes. Annu Rev Genet.

[28] Hentschel U, Steinert M, Hacker J (2000). Common molecular mechanisms of symbiosis and pathogenesis. Trends Microbiol.

[29] Bordenstein SR, Reznikoff WS (2005). Mobile DNA in obligate intracellular bacteria. Nat Rev Microbiol.

[30] Mazel D (2006). Integrons: agents of bacterial evolution. Nat Rev Microbiol.

[31] Valmalette JC, Dombrovsky A, Brat P, Mertz C, Capovilla M, Robichon A (2012). Light- induced electron transfer and ATP synthesis in a carotene synthesizing insect. Sci Rep.

[32] Thao ML, Gullan PJ, Baumann P (2002). Secondary (gamma-Proteobacteria) endosymbionts infect the primary (beta-Proteobacteria) endosymbionts of mealybugs multiple times and coevolve with their hosts. Appl Environ Microbiol.

[33] Moran NA, Jarvik T (2010). Lateral transfer of genes from fungi underlies carotenoid production in aphids. Science.

[34] Nikoh N, Nakabachi A (2009). Aphids acquired symbiotic genes via lateral gene transfer. BMC Biol.

[35] Low DA, Casadesus J (2008). Clocks and switches: bacterial gene regulation by DNA adenine methylation. Curr Opin Microbiol.

[36] Ogden GB, Pratt MJ, Schaechter M (1988). The replicative origin of the E. coli chromosome binds to cell membranes only when hemimethylated. Cell.

[37] Marinus MG, Casadesus J (2009). Roles of DNA adenine methylation in host-pathogen interactions: mismatch repair, transcriptional regulation, and more. FEMS Microbiol Rev.

[38] Low DA, Weyand NJ, Mahan MJ (2001). Roles of DNA adenine methylation in regulating bacterial gene expression and virulence. Infect Immun.

[39] Palmer BR, Marinus MG (1994). The dam and dcm strains of Escherichia coli–a review. Gene.

[40] Wion D, Casadesus J (2006). N6-methyl-adenine: an epigenetic signal for DNA-protein interactions. Nat Rev Microbiol.

[41] Noyer-Weidner M, Trautner TA (1993). Methylation of DNA in prokaryotes. EXS.

[42] Alberts BM (1987). Prokaryotic DNA-replication mechanisms. Philos Trans R Soc Lond B Biol Sci.

[43] Marians KJ (1992). Prokaryotic DNA-replication. Annu Rev Biochem.

[44] Barras F, Marinus MG (1989). The great Gatc - DNA methylation in Escherichia-coli. Trends Genet.

[45] Wu L, Hickson ID (2006). DNA helicases required for homologous recombination and repair of damaged replication forks. Annu Rev Genet.

[46] Hashida SN, Uchiyama T, Martin C, Kishima Y, Sano Y, Mikami T (2006). The temperature-dependent change in methylation of the Antirrhinum transposon Tam3 is controlled by the activity of its transposase. Plant Cell.

[47] Boxem M, van den Heuvel S (2002). *C. elegans* class B synthetic multivulva genes act in G (1) regulation. Curr Biol.

[48] Giangrande PH, Zhu W, Schlisio S, Sun X, Mori S, Gaubatz S, Nevins JR (2004). A role for E2F6 in distinguishing G1/S- and G2/M-specific transcription. Genes Dev.

[49] Cayrol C, Lacroix C, Mathe C, Ecochard V, Ceribelli M, Loreau E, Lazar V, Dessen P, Mantovani R, Aguilar L, Girard JP (2007). The THAP-zinc finger protein THAP1 regulates endothelial cell proliferation through modulation of pRB/E2F cell-cycle target genes. Blood.

[50] Sinzelle L, Izsvak Z, Ivics Z (2009). Molecular domestication of transposable elements: from detrimental parasites to useful host genes. Cell Mol Life Sci.

[51] Bundock P, Hooykaas P (2005). An Arabidopsis hAT-like transposase is essential for plant development. Nature.

[52] Nowacki M, Higgins BP, Maquilan GM, Swart EC, Doak TG, Landweber LF (2009). A functional role for transposases in a large eukaryotic genome. Science.

[53] Roberts D, Hoopes BC, McClure WR, Kleckner N (1985). IS10 transposition is regulated by DNA adenine methylation. Cell.

[54] Top EM, Springael D (2003). The role of mobile genetic elements in bacterial adaptation to xenobiotic organic compounds. Curr Opin Biotechnol.

[55] Blot M (1994). Transposable elements and adaptation of host bacteria. Genetica.

[56] Kucherer C, Lother H, Kolling R, Schauzu MA, Messer W (1986). Regulation of transcription of the chromosomal dnaA gene of *Escherichia coli*. Mol Gen Genet.

[57] Field LM (2000). Methylation and expression of amplified esterase genes in the aphid *Myzus persicae* (Sulzer). Biochem J.

[58] Field LM, Lyko F, Mandrioli M, Prantera G (2004). DNA methylation in insects. Insect Mol Biol.

[59] van Steensel B, Henikoff S (2000). Identification of in vivo DNA targets of chromatin proteins using tethered dam methyltransferase. Nat Biotechnol.

