# Supplementary material for: Adenine methylation may contribute to endosymbiont selection in a clonal aphid population
Source: BMC Genomics. 2014 Nov 19;15(1):999. doi: 10.1186/1471-2164-15-999 (PMC4246565; doi:10.1186/1471-2164-15-999)
Supplement: Supplementary file 1 — Additional file 1: Table S1: Sequences and BLAST results for the adenine methylated fragments. (DOCX 30 KB) [file 12864_2014_6672_MOESM1_ESM.docx]

**Table S1**

| **Methyl-Adenine Fragments** | | | |
| --- | --- | --- | --- |
| **Fragment** | **Sequence** | **Contig No./ Gene identification** | **Sequence identity (amino acid)** |
| **A1** | **GATC**GTGTTATAGAAAATAATGAGAGTGTTAGGCTAAGAGAACCCAAAATGCTCAATAAAGACCGTCTGTTAAGAGATAACCGTTTATGCAAAGCGCTAGTGGGGCTATCGTTGGAAGAATTAAAGACTTTATCCGCTCATTTTTCATCCTGTTATTTAACTTATCGTAAAAATAACCGTGTAGCACATCAACGGAAAATGGGGGCAGGCCAGAAAGGATTTTTACCAACCCCATTGGATAAACTGGTTTTTATTCTGTTGTATTTAAAATGTTATCCGACCTATGACTTACAAGGTTTTCTTTTTGGTTTGGAACGAACCCGAGCTTGTCGCTGGGTGAAATTGCTGTTGCCAGTATTAAGTAGTGTCGTCACGTAATAAAAAATATATTATCATGTAACAAATAACGACAACTGTTGAGATGATTCAATAATGAAAGATGATTCGGGAATGAATTTAGCCCATCGCCGCCACGATATATCC**GATC** | * [ref\|WP_006705102.1\|](http://www.ncbi.nlm.nih.gov/protein/493756248?report=genbank&log$=protalign&blast_rank=1&RID=CBJS80VP01R) hypothetical protein [Candidatus *Regiella insecticola*] | 99% |
|  |  | * [ref\|WP_002757529.1\|](http://www.ncbi.nlm.nih.gov/protein/488845123?report=genbank&log$=protalign&blast_rank=3&RID=CBJS80VP01R) transposase [*Microcystis aeruginosa*] | 43% |
|  |  | *  [ref\|YP_001863818.1\|](http://www.ncbi.nlm.nih.gov/protein/186680622?report=genbank&log$=protalign&blast_rank=7&RID=CBJS80VP01R) transposase, IS4 family protein [*Nostoc punctiforme* PCC 73102] | 41% |
| **A2** | GTGGAGCGGGCTACGACTATAGGCCGATATGGCGGCCGCGGGATTCGATTGGACTCTACTCGACTTTCGGCACTAGGTAATACAAAAACGACCTCCAAAAATGCCCCAAAAACGGTCTGTTTTTATTAAGCAACAGGTCTAATGAACCAAGGAAGTGATAGACCCTGTCCATTTTTATCAGACAAAGTCACATAACTAACCCAGATTTTCATATTAAAGTGACACTCCACCAAGGAAAGCAAGTTCTTTCAAGATATCGTCATCATGAACATGAAAATTTTACGCATTATCTCGAATCACGTTTGCACCAATGGGCTGAATGGTATAGCCGAGGGAATTTTTTTGGCTTAGATTATCCATCCTGCTCTCTAGAATACCGTATCATGACAGAAGGGAATGTATTTCGACGTCCAGGACCTAAGCCCCTGCCCAATCATGAGGCAGCTGAAGAGATAGAATGCTTGGTCAACGAGATAGCACAACAAATCCTACGATGACACGCGCGTTACGGTTCTACTATTTTCAGAGGGGCTCCTTACGTTACAAAGCCAAAAAATTAGCGATTTCTCACACGCAATTCAAATATTATGTCGATATGGCTCATCAATGGTTATTAGGCTGCTTAAGTGGCCGCCATGAAAA**GATC** | * [ref\|WP_006704755.1\|](http://www.ncbi.nlm.nih.gov/protein/493755897?report=genbank&log$=protalign&blast_rank=1&RID=CBGYZU51014) hypothetical protein [Candidatus *Regiella insecticola*] | 77% |
| **A3** | CGGTACGGGCGAGGTGCACTGGCCGCGTGGTGGCCGAGCCGATTGAATGCTATATTGACTGACTATGTTTTTTATTGTTAGTCTGGTCTTAGGTAATATGAGTAGCAAGCAACACCGTCAAAGCAGTGAGTGGGAAAATCTGAGTCAACCAGCTAAAGATGAGAAAATAAGCGCGCCTCCAAAACCGAGCAACGATATCCCTCAGTGACAAGCAAGTCGTTATCTTTGTATCGATGAGCACAAGAGGTTTAGGACAGGCTCTAATGATTATCTTCAGTACCGAGGTGGTGAAATTGGTAGACACGCTACCTTGAGGTGGTAGTGCTCGATTGGGCTTACGGGTTCAAATCCCGTCCTCGGTACCAATTCATAGAGATAACTTGCTTTTGGATGAT**GATC** | *  [ref\|WP_006704986.1\|](http://www.ncbi.nlm.nih.gov/protein/493756130?report=genbank&log$=protalign&blast_rank=1&RID=CBKAME0W014) preprotein translocase subunit SecG [Candidatus *Regiella insecticola*] | 96% |
|  |  | * [ref\|WP_004720391.1\|](http://www.ncbi.nlm.nih.gov/protein/490858349?report=genbank&log$=protalign&blast_rank=3&RID=CBKAME0W014) preprotein translocase subunit SecG [*Yersinia ruckeri*] | 63% |
| **A4** | **GATC**ACACCAACCGTGCTGTCGAGCAAAGCATCAAGACAAGCTGGCAATGGACTCAGCCGATTTTTAATACCCTCTTTACACTAGCAGCAAAAAGAAGAGGCGATAATTACAGTATATTCCTCAATACGGCTATCGCTTTTAATCAAATCAATCTCAATATTGATAATACCTACAGCAATAAACAATTCACTACCAGTGCACAATATAAACAACAGCGAGTTGATGACTATGGCGCTACCATTTTTGGTGTCGACAGTGATATCACTGGAAAAAATTACCATTTCAGCAGTGATATTCAACGCTTAGGTAGCCGAGGGAATAGCTGGCTACGCATCGGCATCAATGACCAAGT**GATC**TTTTTCACACTAAACAACATGTAATCACAAACACAAACCAAGTGCTTATCTTTTAGTCAAAAAGTGACCGAGTTCCTTTTCAGCACCAGATGATATTCCGCTTCCTCGAACGGCCTTCGCTCTGCTCAGGCCATTCTTGCGGCGAGCGTTACC**GATC** | *  [ref\|WP_002231932.1\|](http://www.ncbi.nlm.nih.gov/protein/488160724?report=genbank&log$=protalign&blast_rank=1&RID=CBMMFNY9015) hypothetical protein [*Yersinia pestis*] | 35% |
|  |  | *  [ref\|WP_006707270.1\|](http://www.ncbi.nlm.nih.gov/protein/493758493?report=genbank&log$=protalign&blast_rank=34&RID=CBMMFNY9015) hypothetical protein [*Candidatus* *Regiella insecticola*] | 89% (14% coverage) |
| **A5** | CCTGCCGCCATAGCAATCGCCAATGAGCTGCTGGGGAAAATCAGTGAGCATGTGGAGAAAGGAGAGATAAAACCGGCACTGTGGTCATCGCTGCGCAATGTCTCTGCCGGTCATTTGGCGCGTCTCGAAAAATTCAGCCACCAGGGTGGCAGGCTGATGCAAGGCTATGGCTACACTCGCGGGATACAG**GATC** | *  [ref\|WP_006706710.1\|](http://www.ncbi.nlm.nih.gov/protein/493757915?report=genbank&log$=protalign&blast_rank=1&RID=CBMZ81XK01R) Peptidase C80 family, partial [*Candidatus Regiella insecticola*] | 81% |
|  |  | *  [ref\|WP_006706916.1\|](http://www.ncbi.nlm.nih.gov/protein/493758127?report=genbank&log$=protalign&blast_rank=2&RID=CBMZ81XK01R) RTX toxin [*Candidatus Regiella insecticola*] | 49% |
| **A6** | **GATC**CTAGTGCAGCAATACAGCATTTGCGCAATACGGTGGATTATGAAAGCTGGTTATATGAAATCTCACCCAGTCCGCTCGCGGCAGAAATGCGGATGAAAAATGTCAACTTATTATTTCTCTGGCTGGAGGAAATGCTGTCCGGCTCTGCATTGCATGAACCCATGACATTAATACAGGCGGTCACTCGTTTTACTCTGCGCGACCTAATGGAAAAAGGAAAGAATAATAAAGAAGAGGATGAAGTACAGCTGATGACACTACATGCTTCTAAAGGGCTGGAATTTCCCTATGTATTTTTAGTTGGCATGGAAGAAGGATTATTACCACATCAAAATAGCCTCGATGAAGGCCATCTCAATGAAGGTAATGTAGATGAAGAAAGGCGTTTGGCTTATGTCGGCATTACTCGTGCTCAGCACGAACTCTTTTTTACCTTATGTAAAGAACGACGTCAGTACGGTGAATTAATCCGCCCAGAGCCTAGCCGTTTTTTGTTAGAGTTGCCGCAAGAT**GATC** | *  [ref\|WP_006705384.1\|](http://www.ncbi.nlm.nih.gov/protein/493756544?report=genbank&log$=protalign&blast_rank=1&RID=CBN7M0BB01R) ATP-dependent DNA helicase Rep [*Candidatus Regiella insecticola*] | 94% |
|  |  | * [emb\|CBX70162.1\|](http://www.ncbi.nlm.nih.gov/protein/330859830?report=genbank&log$=protalign&blast_rank=2&RID=CBN7M0BB01R) ATP-dependent DNA helicase rep [*Yersinia enterocolitica* W22703] | 78% |
|  |  | *  [ref\|WP_023242920.1\|](http://www.ncbi.nlm.nih.gov/protein/555260761?report=genbank&log$=protalign&blast_rank=6&RID=CBN7M0BB01R) ATP-dependent DNA helicase Rep, partial [*Salmonella enterica*] | 74% |
