# Supplementary material for: Adenine methylation may contribute to endosymbiont selection in a clonal aphid population
Source: BMC Genomics. 2014 Nov 19;15(1):999. doi: 10.1186/1471-2164-15-999 (PMC4246565; doi:10.1186/1471-2164-15-999)
Supplement: Supplementary file 2 — Additional file 2: Figure S1: The nucleotides sequence and amino acid prediction of the aphid Transposase gene (A1 fragment) The DNA fragment (A1) was isolated from A. pisum based on methylation of adenine. (a) The nucleotide sequence of the original DNA fragment and the deduced amino acid encodes for a transposase gene. The translated sequence matches the transposase of: Microcystis aeruginosa (accession number WP_002757529.1) and Nostoc punctiforme (accession number YP_001863818.1). In gray the two putative sites for methylation on adenine flanking the region. (b) The EST-CV840801, represents a DNA fragment from the A. pisum EST data bank that shows high sequence similarity to the A1 DNA fragments. The upper lines are the nucleotide sequence encoding for transposase, and below is the deduced amino-acid residues. In bold, the transposase coding region. Mark in gray, the three putative sites for methylation on adenine that were identified upstream to the transposase gene. The primers sequences underlined and labeled in bold. (DOC 42 KB) [file 12864_2014_6672_MOESM2_ESM.doc]

**Figure S1**

**A**

1 GATCGTGTTATAGAAAATAATGAGAGTGTTAGGCTAAGAGAACCCAAAATGCTC

1 **D R V I E N N E S V R L R E P K** M **L**

55 AATAAAGACCGTCTGTTAAGAGATAACCGTTTATGCAAAGCGCTAGTGGGGCTA

19 **N K D R L L R D N R L C K A L V G L**

109 TCGTTGGAAGAATTAAAGACTTTATCCGCTCATTTTTCATCCTGTTATTTAACT

37 **S L E E L K T L S A H F S S C Y L T**

163 TATCGTAAAAATAACCGTGTAGCACATCAACGGAAAATGGGGGCAGGCCAGAAA

Transposase

55 **Y R K N N R V A H Q R K M G A G Q K**

217 GGATTTTTACCAACCCCATTGGATAAACTGGTTTTTATTCTGTTGTATTTAAAA

73 **G F L P T P L D K L V F I L L Y L K**

271 TGTTATCCGACCTATGACTTACAAGGTTTTCTTTTTGGTTTGGAACGAACCCGA

91 **C Y P T Y D L Q G F L F G L E R T R**

325 GCTTGTCGCTGGGTGAAATTGCTGTTGCCAGTATTAAGTAGTGTCGTCACGTAA

109 **A C R W V K L L L P V L S S V V T** *

379 TAAAAAATATATTATCATGTAACAAATAACGACAACTGTTGAGATGATTCAATA

433 ATGAAAGATGATTCGGGAATGAATTTAGCCCATCGCCGCCACGATATATCCGAT

487 C

**B**

2 tca agg gaa gaa atc ttt ggt caa gaa cgt gaa ctc aca ttt aaa 46

47 tat ctg aca ccg **gat tat cat ggc agc gca ta**c cac agt gat gat 91

92 caa tac tat act aat tta agc cca ttt acc gtt caa caa cga gag 136

137 att act caa tct att ctc gat gaa att gca gat cta acg gg**t tta** 181

182 **aga ttc cgc ctg gtt g**ga agc gat gac gca agt aat tta acc ttt 226

227 aaa cga gtt gct taa taa gcc tat tgc att ttc gat cgt gtt ata 271

* * A Y C I F D R V I 90

272 gaa aat aat gag agt gtt agg cta aga gaa ccc aaa aag ctc aat 316

91 E N N E S V R L R E P K K L N 105

317 aaa gac cgt ctg tta aga gat aac cgt tta tgc aaa gcg c**ta gtg** 361

106 K D R  **L L R D N R L C K A L V**  120

362 **ggg cta tcg ttg gaa** gaa tta aag act tta tcc gct cat ttt tca 406

121 **G L S L E E L K T L S A H F S** 135

407 tcc tgt tat tta act tat cgt aaa aat aac cgt gta gca cat caa 451

136 **S C Y L T Y R K N N R V A H Q** 150

Transposase

452 cgg aaa atg ggg gca ggc cag aaa gga ttt tta cca acc cca ttg 496

151 **R K M G A G Q K G F L P T P L** 165

497 gat aaa ctg gtt ttt att ctg ttg tat tta aaa tgt tat ccg acc 541

166 **D K L V F I L L Y L K C Y P T** 180

542 tat gac tta caa ggt ttt ctt ttt ggt ttg gaa cga acc cga gct 586

181 **Y D L Q G F L F G L E R T R A** 195

587 tgt cgc tgg gtg aaa ttg ctg ttg cca gta tta gag atg acg ttg 631

196 **C R W V K L L L P V L E M T L** 210

632 gga cat gaa tgt gtg tta ccg gca cgt caa atc cgt tcc atg gag 676

211 **G H E C V L P A R Q I R S M E** 225

677 gag ttt tgc cac gct ttt ccg ggt gtt cgt gac gtt ttt att gat 721

226 **E F C H A F P G V R D V F I D** 240

722 ggg aca gaa cgt cct gtt caa aag cct aaa aat acc cga cgc cgt 766

241 **G T E R P V Q K P K N T R R R** 255

767 aac aaa atg tac tcg gga aag aag aga cag act acc ggc aaa gtg 811

256 **N K M Y S G K K R Q T T G K V** 270

812 gtc atg atg act gac gaa aca agg cga gtg ggt ttt ctc tca ctg 856

271 **V M M T D E T R R V G F L S L** 285

857 agt **aaa aat ggt cga cgt cat gac a**aa cgc 886

286 **S K N G R R H D K R**
