# Supplementary figures and images for: Adenine methylation may contribute to endosymbiont selection in a clonal aphid population
Source: BMC Genomics. 2014 Nov 19;15(1):999. doi: 10.1186/1471-2164-15-999 (PMC4246565; doi:10.1186/1471-2164-15-999)

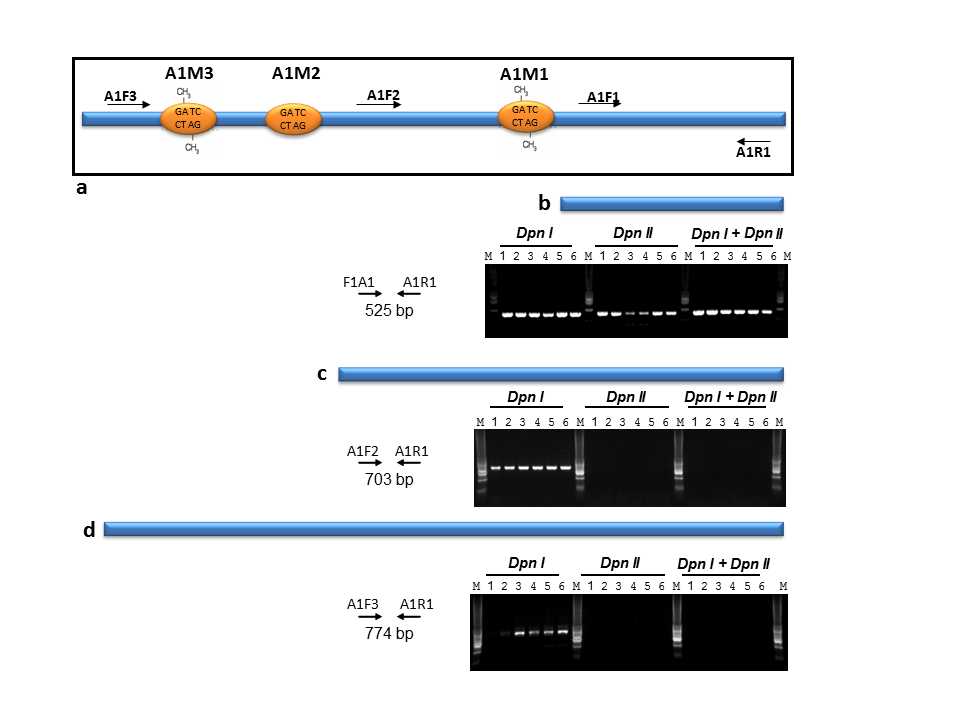

Supplement: Supplementary file 3 — Additional file 3: Figure S2: Extended analysis of adenine methylation in the transposase gene. Total DNA was extracted from each individual A. pisum aphid propagated at 20°C (orange variant, lanes 1–3) or at 8°C (green variant, lanes 4–6). The obtained gDNA was pre-digested with EcoRI, and then re-digested by DpnI, DpnII or both. (a) Schematic representation of the extended A1 fragment (transposase gene). Orange circles represent the potential adenine methylation sites and arrows indicate the primers positions. The digested gDNA was amplified by PCR using three pairs of primer: A1R1 combined with (b) A1F1, (c) A1F2 or (d) A1F3. (TIFF 122 KB) [file 12864_2014_6672_MOESM3_ESM.tiff]

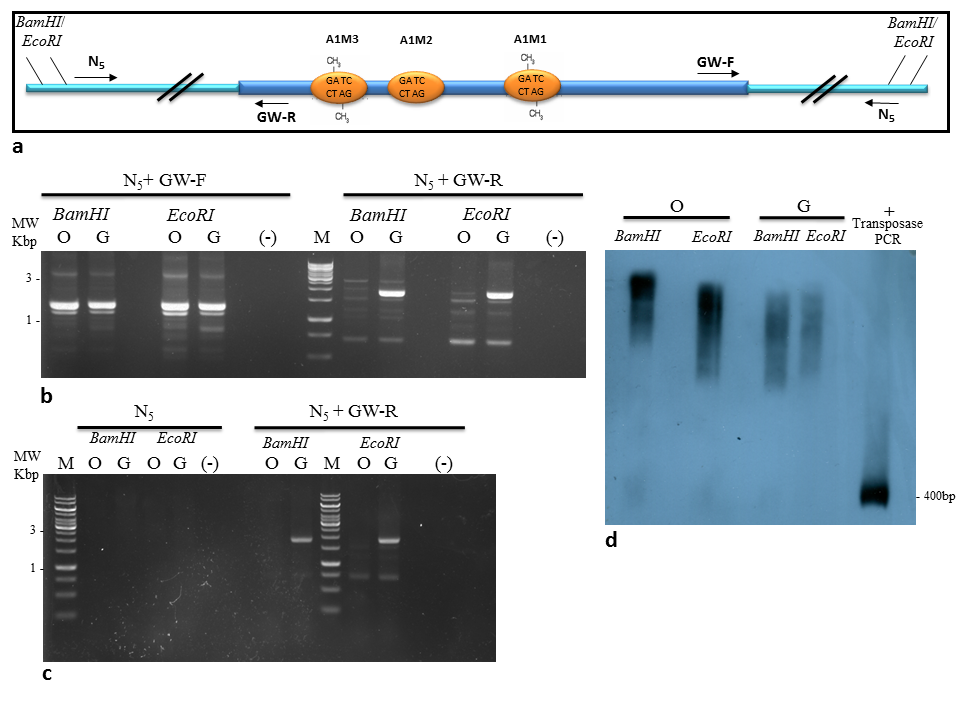

Supplement: Supplementary file 4 — Additional file 4: Figure S3: Genome rearrangement. (a) Schematic representation of the extended A1 fragment (transposase gene) and the method used. Restriction enzymes indicated at the top, arrows indicates primer position and orientation. Orange circles represent the potential adenine methylation sites (A1M1-A1M3). (b) Differential amplification pattern on digested gDNA obtained from orange (O) and green (G) aphids. PCRs carried out using singular transposase specific primer [GW-F (left panel) or GW-R (right panel)] combined with random hexamer (N5), (-) indicates negative control, without template. (c) Controls: random hexamer solely (left panel) and increasing the annealing temperature in order to increase the specificity of the transposase specific primer (right panel). (d) Southern blot analysis on orange or green aphid variants digested gDNA (EcoRI or BamHI), the transposase DIG-probe (400 bp) reacted positively with the unlabeled amplicon (positive control, right lane). (TIFF 357 KB) [file 12864_2014_6672_MOESM4_ESM.tiff]
